# Getting it Right the First Time: Frozen Sections for Diagnosing Necrotizing Soft Tissue Infections

**DOI:** 10.1007/s00268-020-05786-7

**Published:** 2020-09-29

**Authors:** Femke Nawijn, Falco Hietbrink, Marijke R. van Dijk

**Affiliations:** 1 Department of Surgery, University Medical Center Utrecht, Utrecht, The Netherlands; 2 Department of Pathology, University Medical Center Utrecht, Utrecht, The Netherlands

## Abstract

**Background:**

The aim of this study was to investigate which histopathologic findings are most indicative for necrotizing soft tissue infections (NSTIs) in ambivalent cases.

**Methods:**

Patients undergoing surgical exploration for suspected NSTIs with obtainment of incisional biopsies for histopathological assessment were included from January 2013 until August 2019. The frozen sections and formalin-fixed paraffin-embedded (FFPE) samples were retrospectively re-assessed. The primary outcome was the discharge diagnosis.

**Results:**

Twenty-seven (69%) biopsies of the 39 included samples were from patients with NSTIs. Microscopic bullae (*p* = 0.043), severe fascial inflammation (*p* < 0.001) and fascial necrosis (*p* < 0.001) were significantly more often present in the NSTI group compared to the non-NSTI group. Muscle edema (*n* = 5), severe muscle inflammation (*n* = 5), muscle necrosis (*n* = 8), thrombosis (*n* = 10) and vasculitis (*n* = 5) were most frequently only seen in the NSTI group. In thirteen tissues samples, there were some discrepancies between the severity of findings in the frozen section and the FFPE samples. None of these discrepancies resulted in a different diagnosis or treatment strategy.

**Conclusion:**

Microscopic bullae, severe fascial or muscle inflammation, fascial or muscle necrosis, muscle edema, thrombosis and vasculitis upon histopathological evaluation all indicate a high probability of a NSTI. At our institution, diagnosing NSTIs is aided by using intra-operative frozen section as part of triple diagnostics in ambivalent cases. Based on the relation between histopathologic findings and final presence of NSTI, we recommend frozen section for diagnosing NSTIs in ambivalent cases.

## Introduction

Early diagnosis and immediate radical surgical treatment are vital for reducing the mortality rate of necrotizing soft tissue infections (NSTIs) [1–3]. NSTIs are notorious for being difficult to diagnose based on clinical symptoms, resulting in high rates of misdiagnosis and treatment delay [4, 5]. To resolve this problem, the approach using triple diagnostics (diagnosis based on macroscopic, histopathologic and microbiologic findings) has been proposed for ambivalent macroscopic cases [6]. The intra-operative evaluation of frozen sections and Gram stains enables identification of microscopic signs of NSTIs [6]. The use of triple diagnostics demonstrated a relatively low mortality rate and shorter intensive care stays, indicating a less severe disease course, likely due to identification of the NSTI in its earlier stages when only microscopically signs are visible [7]. However, there is still no clear consensus concerning the use of frozen sections for diagnosing NSTIs [8, 9]. This is mainly caused by the lack of current literature on this topic [10–13]. Back in 1984, Stamenkovic et al. [11] first reported that frozen sections might reduce the mortality rate of NSTIs resulting from the earlier recognition of the infection. However, the first guideline recommendation for using frozen sections to diagnose NSTIs, as part of triple diagnostics, was not made until 2018 [8]. Nonetheless, this guideline also stated that frozen sections are not very practical and require availability and experience of the pathologists, while intra-operative assessment of frozen sections has already become routine practice in surgical oncology [8, 14]. Therefore, the aim of this study was to investigate which histopathologic findings are most indicative for the diagnosis NSTI.

## Methods

### Study design

Patients undergoing surgical exploration for suspected NSTIs with obtainment of tissue biopsies for histopathological assessment during this initial exploration at an academic hospital were prospectively identified from January 2013 until August 2019 and included in this study. Biopsies taken secondarily from patients with an already confirmed and current NSTI were excluded. The clinical outcomes of patients included up to January 2019 were previously reported in an article by Nawijn et al. [7]. NSTIs were diagnosed based on macroscopic findings of necrosis of the subcutaneous tissue, fascia or muscle during surgical exploration. In case of ambivalent macroscopic findings such as fascial edema without clear necrosis, the triple diagnostics algorithm was used. Using this algorithm, the diagnosis NSTI was confirmed if either the intra-operative assessed frozen section or Gram stain was positive. If both were negative, the diagnosis NSTI was rejected [6]. Final diagnosis was made by clinical follow-up. When triple diagnostics were utilized, the following histopathologic characteristics of NSTIs were often used for assessing tissue biopsies: necrosis of superficial fascia, polymorphonuclear infiltration of the deep dermis and fascia, fibrinous thrombi of arteries and veins passing through the fascia, angiitis with fibrinoid necrosis of vessels walls and micro-organisms within the destroyed fascia and dermis [6, 11].

### Data collection and outcome measures

**Fig. 1:**
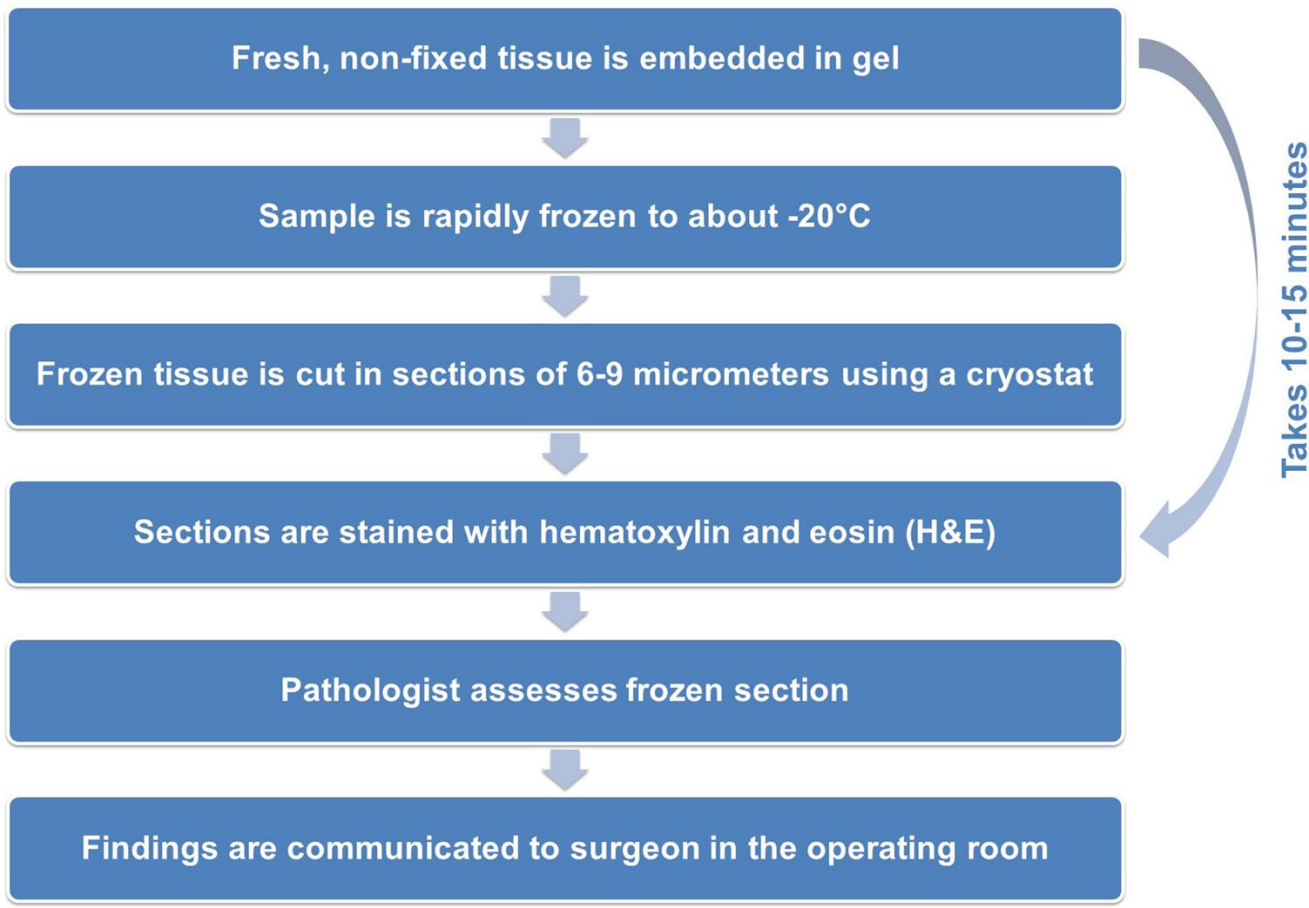
Flowchart of sampling process of frozen sections

Data collected from the medical charts included physical examination findings, intra-operative findings (based on surgical notes), histopathologic findings (both frozen sections and standard formalin-fixed paraffin-embedded (FFPE) samples) and discharge diagnosis. The sampling process for frozen sections is detailed in Fig. 1 [6]. At our institute, all samples (frozen and FFPE) are archived for 35 years and all tissue blocks from which sections were sliced are archived for 110 years. A data-collection form was designed to retrospectively re-assess the frozen sections in a protocolized manure without knowing the conclusion given by the previous pathologist (Appendix 1). Notion was made of which tissue layers were available and if they showed any abnormalities. All samples were assessed by an experienced pathologist. The frozen section, if available, was examined prior to assessing the FFPE sample. For the assessment of the overall histopathological characteristics of NSTIs, the most atypical finding from either the frozen section or FFPE sample was recorded. The primary outcome of this study was the discharge diagnosis reported in the discharge papers.

### Statistical analyses

Categorical variables are presented as fractions or frequencies. Missing data were handled using pairwise deletion. The Fisher exact test was used for dichotomous independent variables and the *Χ*^2^ for trend for ordinal independent variables. For all analyses, a *p* value < 0.05 was considered statistically significant. Data were analyzed using STATA (StataCorp. 2013. Stata Statistical Software: Release 13. College Station, TX: StataCorp LP).

## Results

**Fig. 2:**
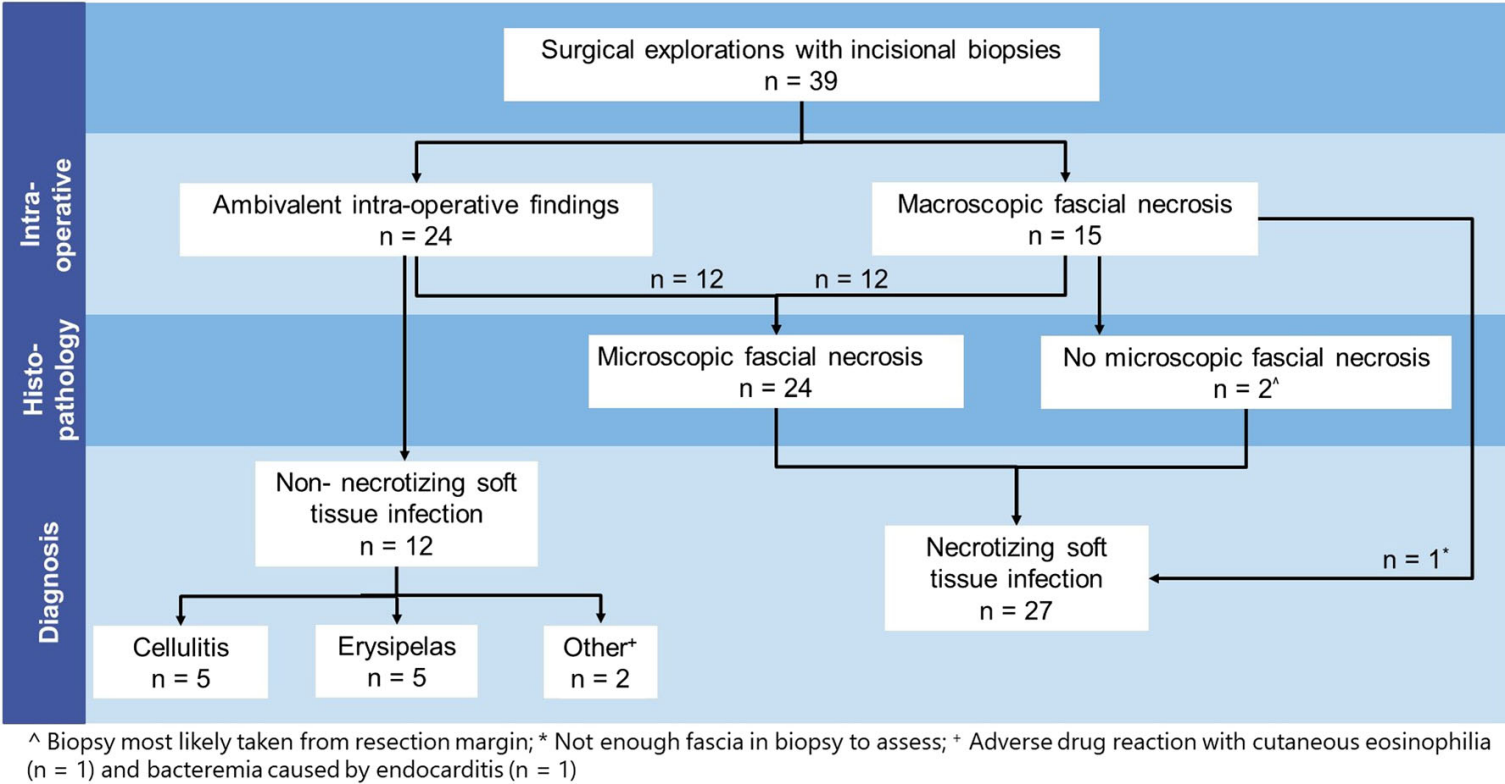
Flowchart of patient undergoing surgical exploration for suspected necrotizing soft tissue infection with obtainment of biopsies for histopathological assessment

**Fig. 3:**
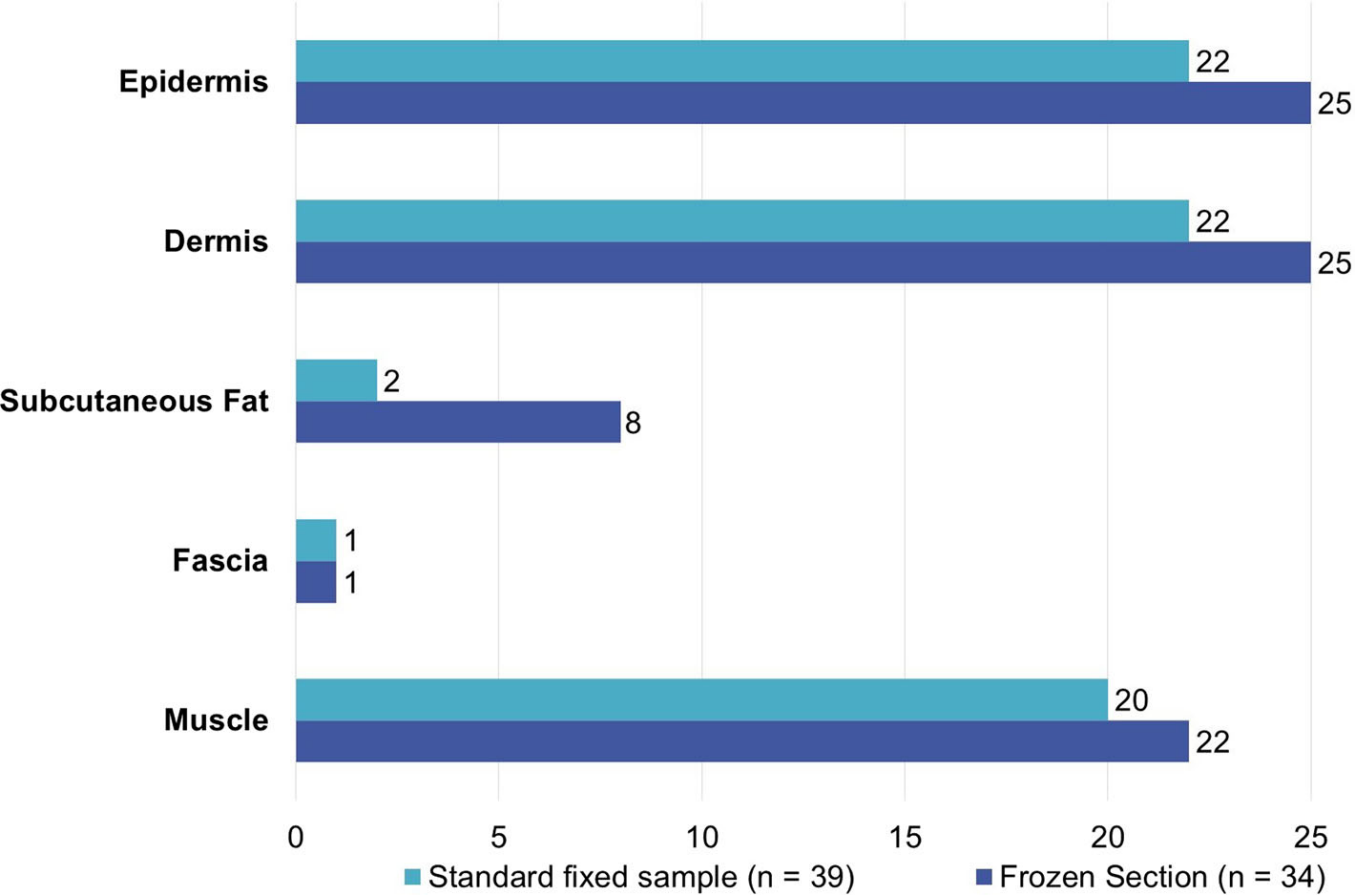
Tissue layers absent for histopathologic assessment in incisional biopsies obtained from patients with suspected necrotizing soft tissue infections

Thirty-seven patients underwent surgical explorations during which biopsies were taken for histopathologic evaluation. Twenty-six patients were diagnosed with NSTIs (70%), of which four patients eventually died (15%). One patient had initially a negative exploration; however, due to clinically deterioration triple diagnostics were repeated two days later and were positive for NSTI. Another patient underwent a new exploration three months after hospitalization for an earlier NSTI, which was eventually negative (diagnosis: erysipelas). From both patients, the histopathologic samples were included, resulting in a total of 39 biopsies from 37 patients (Fig. 2). Eight biopsies were truly full-thickness biopsies (epidermis to muscle). The epidermis and dermis were both most commonly missing (both in 22 samples) (Fig. 3).

### Histopathologic characteristics of NSTIs

**Fig. 4:**
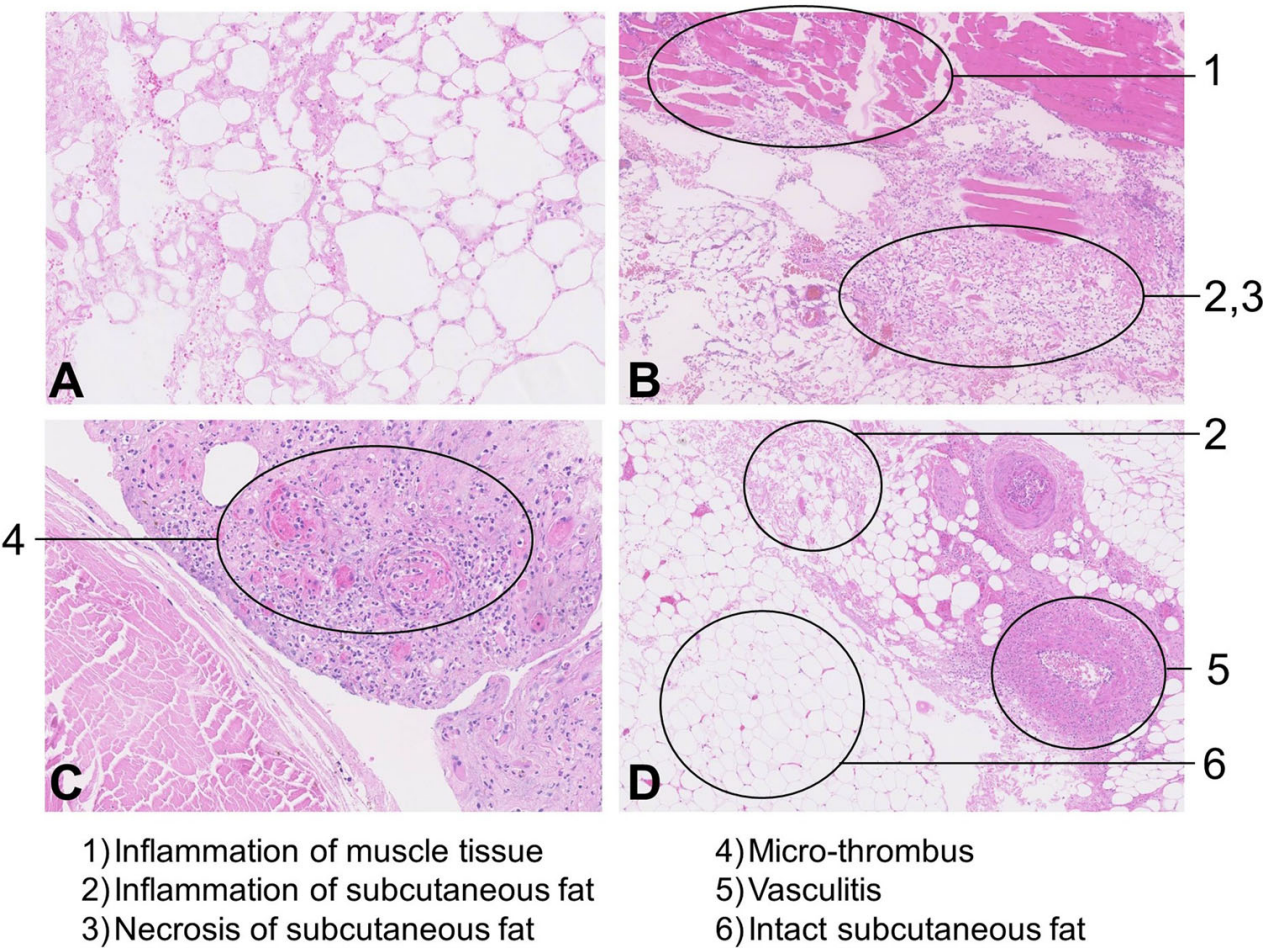
Microscopic views of different histopathologic findings in necrotizing soft tissue infections. **a** Necrotic subcutaneous fat; **b** inflammation of subcutaneous fat and muscle tissue, combined with necrosis of the subcutaneous fat; **c** micro-thrombi; **d** necrotic and intact subcutaneous fat combined with vasculitis

**Fig. 5:**
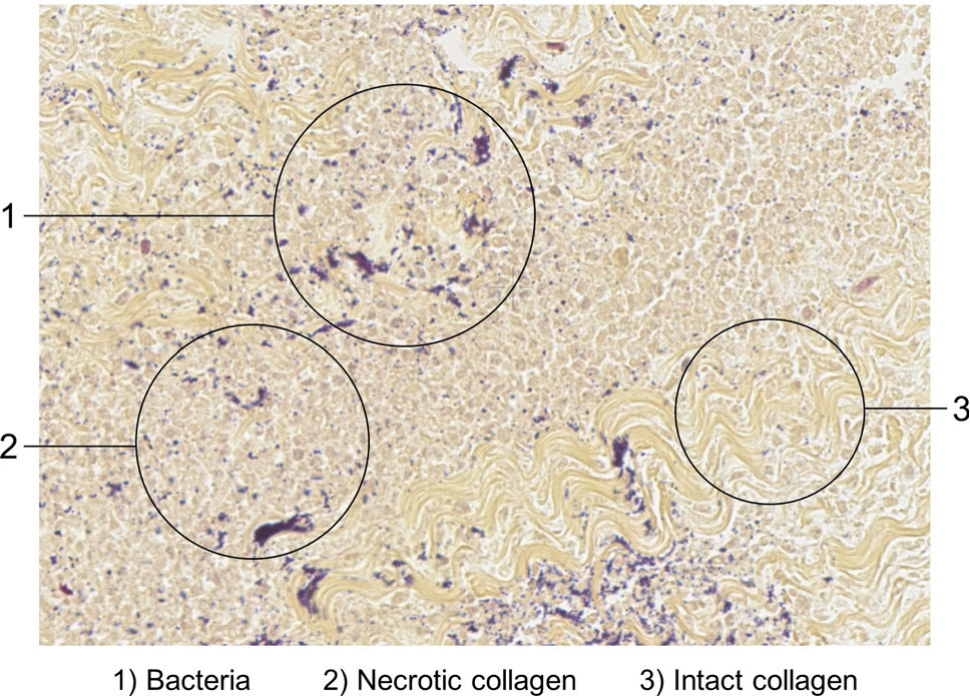
Microscopic view after Gram stain of biopsy from patient with necrotizing soft tissue infection showing multiple bacteria surrounded by intact and necrotic collagen

**Fig. 6:**
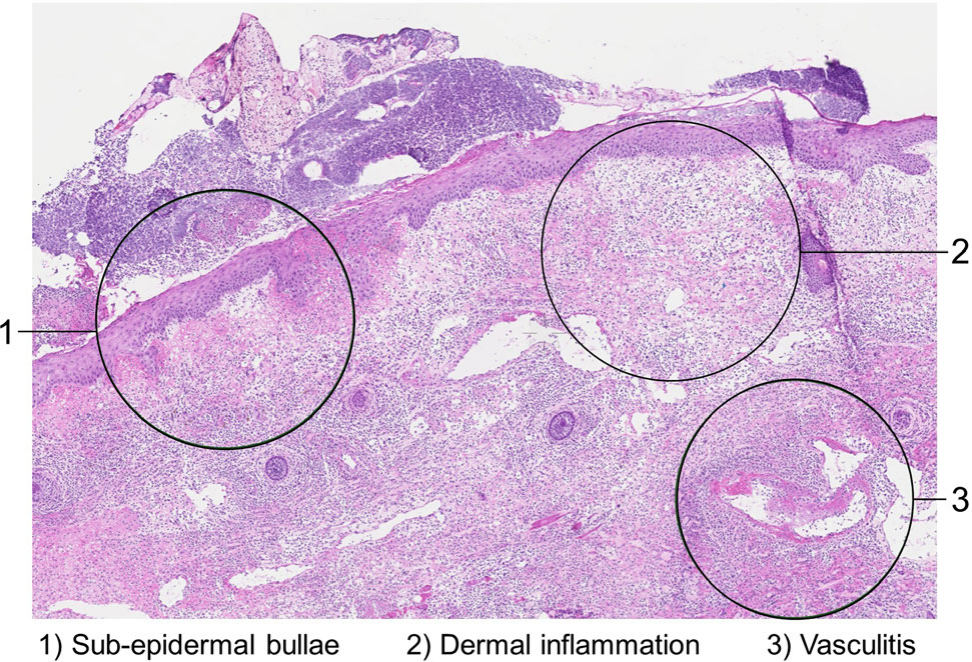
Microscopic view of frozen section from patient with necrotizing soft tissue infection showing sub-epidermal bullae, severe dermal inflammation and vasculitis

**Fig. 7:**
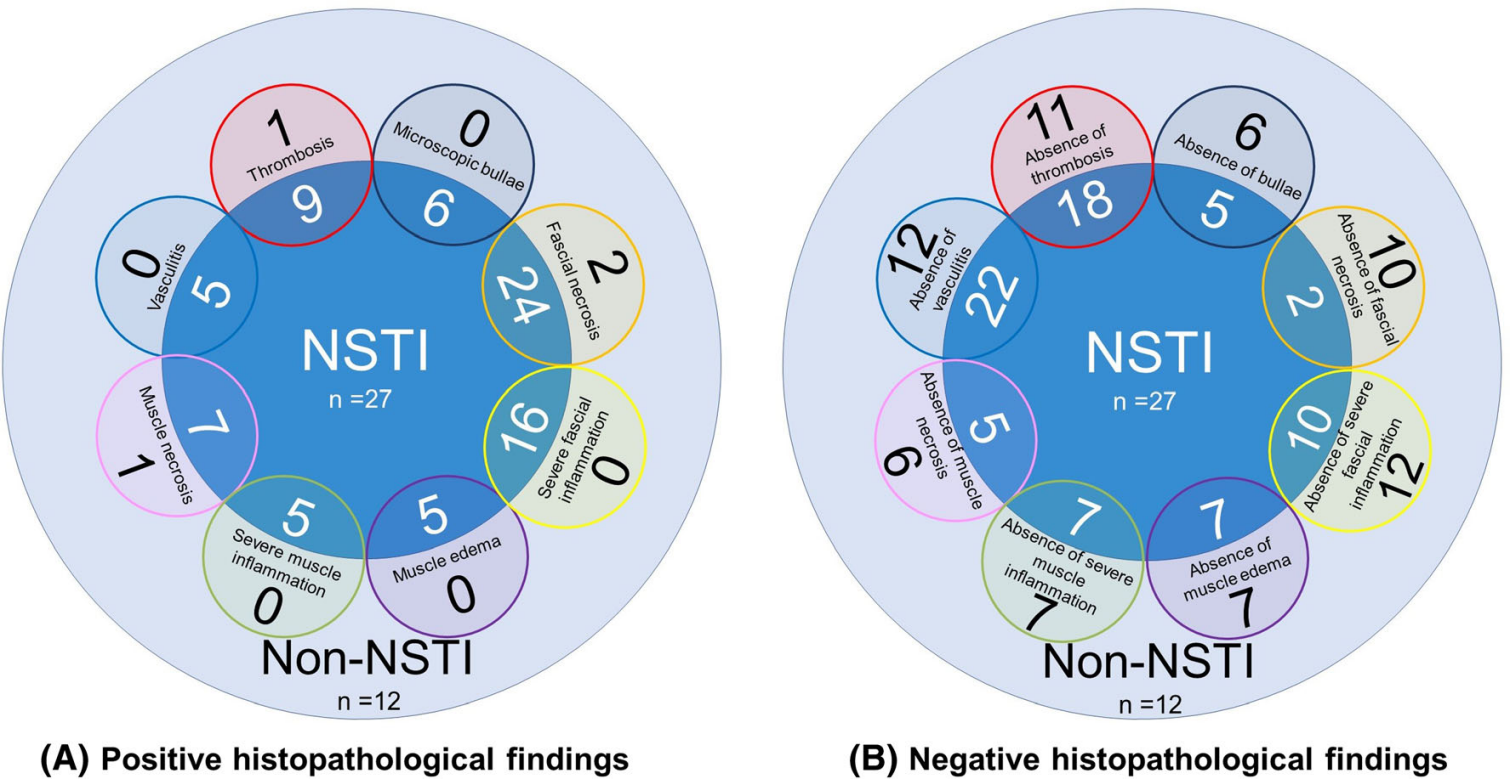
Histopathological findings of necrotizing soft tissue infection (NSTI) found in biopsies from patients undergoing surgical exploration for suspected NSTI. **a** Positive histopathological findings. **b** Negative histopathological findings Figure legend: Dark blue inner circle represents all 27 NSTI patients; white numbers report the number of NSTI patients with a certain (in case of **b**: absent) histopathological findings (written in black); light blue outer circle represents all 12 non-NSTI patients; black numbers report the number of non-NSTI patients with a certain (in case of **b**: absent) histopathological findings (written in black)

Bullae (*p* = 0.043), more severe fascial inflammation (*p* < 0.001), especially if rated as severe inflammation (*p* < 0.001) and fascial necrosis (*p* < 0.001) were significantly more often present in the NSTI group compared to the non-NSTI group (Table 1; Figs. 4, 5, 6). There were two NSTI patients without microscopic fascial necrosis; however, both had reported macroscopic fascial necrosis. Microscopic muscle edema (*n *= 5), severe muscle inflammation (*n* = 5), muscle necrosis (*n* = 8), thrombosis (*n* = 10) or vasculitis (*n* = 5) were in a minority of the cases found; however if present, it was in all but two case only found in NSTI patients (Fig. 7a).

**Table 1:** Histopathological characteristics of incisional biopsies taken from patients with suspected necrotizing soft tissue infections

Tissue layer	NSTI (*n* = 27)	Non-NSTI (*n* = 12)	*p* value
Epidermis (*n* = 17)			
No abnormalities	4/11	5/6	0.131^a^
Bullae	6/11	0/6	**0.043**^a^
Necrosis	1/11	1/6	1.000^a^
Dermis (*n* = 17)			
No abnormalities	2/11	1/6	1.000^a^
Edema	8/11	5/6	1.000^a^
Acute inflammation			1.000^b^
Mild	1/11	3/6	
Moderate	1/11	1/6	
Severe	3/11	0/6	
Necrosis	1/11	0/6	1.000^a^
Subcutaneous fat (*n* = 37)			
Inflammation (regardless of severity)	18/26	7/11	1.000^a^
Necrosis	20/26	5/11	0.122^a^
Fascia (*n* = 38)*			
No abnormalities	0/26	3/12	**0.026**^a^
Edema	13/26	8/12	0.486^a^
Acute inflammation			** < 0.001**^b^
Mild	1/26	5/12	
Moderate	6/26	2/12	
Severe	16/26	0/12	
Necrosis	24/26	2/12^+^	** < 0.001**^a^
Muscle (*n* = 19)			
No abnormalities	0/12	3/7	**0.036**^a^
Edema	5/12	0/7	0.106^a^
Acute inflammation			0.160^b^
Mild	1/12	3/7	
Moderate	1/12	0/7	
Severe	5/12	0/7	
Necrosis	7/12	1/7	0.147^a^
Vessels (*n* = 39)			
Thrombosis	9/27	1/12	0.131^a^
Vasculitis	5/27	0/12	0.299^a^

NSTI = Necrotizing Soft Tissue Infection; * Not enough fascia to assess (*n* = 1); ^+^ hypothesized to be caused due to systemic (micro)vascular disease in patient with adverse drug reaction with cutaneous eosinophilia and early abscess formation in patient with cellulitis; ^a^Fisher exact test used; ^b^*Χ*^2^ for trend used. Values in bold denote statistically significant result

### Histopathologic characteristics of non-NSTIs

Twelve biopsies were from patients with eventually a non-NSTI discharge diagnosis. Severe fascial inflammation (*n* = 12), vasculitis (*n* = 12), thrombosis (*n* = 11) and fascial necrosis (*n* = 10) were absent in all or a majority of the cases (Fig. 7b). If histopathological abnormalities were found, this was most often either edema in the dermis (5/6), subcutaneous fat inflammation (7/11) or fascial edema (8/12) (Table 7, Appendix 2). These patients had as discharge diagnosis either cellulitis (*n *= 5), erysipelas (*n* = 5), bacteremia with septic embolisms caused by an endocarditis (*n* = 1) or an adverse drug reaction (ADR) with cutaneous eosinophilia (*n* = 1). One of the two non-NSTI patients with fascial necrosis also had microscopic thrombi (diagnosis: ADR with cutaneous eosinophilia). The thrombi and fascial necrosis were most likely secondary to the patient’s systemic (micro)vascular disease, which also explained the secondary influx of neutrophils. In the other patient with microscopic fascial necrosis (diagnosis: cellulitis), the necrosis was found during re-assessment of the biopsies for this study and was not reported by the pathologist that originally assessed the biopsy. This pathologist concluded ambivalent signs of a NSTI in the frozen section. Combined with a negative Gram stain and macroscopic vital fascia, the diagnosis NSTI was found unlikely and no debridement was performed. This patient did not clinically deteriorate after the exploration, however developed an abscess at the same location a week later. In retrospect, it is hypothesized that early abscess formation might have caused the necrosis seen microscopically.

### Discrepancies

Thirty-four biopsies were first processed as frozen section followed by processing as FFPE sample. The five other samples were only processed as FFPE samples. In eleven samples (out of 34), one or more tissue layers were missing in the frozen section compared to the FFPE sample. Most often, the subcutaneous fat tissue was missing in the frozen section but present in the FFPE sample (*n* = 6) (Fig. 3). In thirteen samples (out of 34), there was a discrepancy between the severity of findings upon histopathological assessment of the frozen section and the FFPE samples (Table 2). However, none of these less severe findings in the frozen sections resulted in a different diagnosis or treatment strategy. In eleven of those thirteen samples, material of the FFPE sample was sliced from the same biopsy as the frozen section, and in the two other samples, the material of the sample was sliced from additionally taken tissue biopsies from which no frozen section was made.

**Table 2:** Histopathological discrepancy between frozen sections and formalin-fixed paraffin-embedded samples from patients with suspected necrotizing soft tissue infections

Reason for discrepancy	*n* = 13
Inflammation less severe in frozen section	5
No thrombi in frozen section	4
Inflammation more severe in frozen section	1
Inflammation less severe in frozen section AND no muscle necrosis in frozen section	1
Inflammation less severe in frozen section AND no thrombi in frozen section	1
No fascial necrosis in frozen section AND no vasculitis in frozen section	1

### Physical examination

The findings upon physical examination prior to surgery were reported in 38 cases. Blue or purple skin discoloration was reported in nineteen cases (15 in NSTI group). All of these fifteen NSTI cases had either microscopic necrosis of the subcutis or fascia, and in five cases, microscopic thrombi were found.

Macroscopic bullae were reported in eleven cases (9 in NSTI group). Microscopic bullae were as well seen upon histopathologic evaluation in five NSTI cases and only microscopically seen in one case (not reported upon physical examination). In the two non-NSTI cases with macroscopic bullae, the discharge diagnoses were a bacteremia caused by an endocarditis and an ADR with cutaneous eosinophilia.

### Intra-operative findings

In 24 cases (out of 39), no macroscopic fascial necrosis was visible upon surgical exploration and triple diagnostics were necessary to make the diagnosis NSTI more or less likely. In 12 out of those 24 cases, microscopic necrosis of the fascia was found (Fig. 2). In five out of the twelve NSTI cases without macroscopic necrosis, fascial edema was also not explicitly reported. In total, fascial edema was explicitly reported in fourteen out of the 39 cases, of which two cases were non-NSTIs (diagnosis: both erysipelas).

## Discussion

The use of intra-operative frozen section as part of the triple diagnostics algorithm in less evident macroscopic cases of NSTI has been suggested. Based on our findings, we propose major and minor histopathologic criteria for patients with suspected NSTIs (Table 3) and provide key notes for using frozen section sections (Table 4). In case of using frozen sections, pathologist should especially look for bullae, muscle edema, severe fascial or muscle inflammation, fascial or muscle necrosis and vascular abnormalities (e.g. thrombosis or vasculitis). The major criteria are the significant findings from this study; the minor criteria are findings that were distinctly seen in patients with NSTIs. These findings are in our opinion beneficial for recognizing NSTIs in frozen sections and should be the focus of larger studies with sufficient power.

**Table 3:** Major and minor histopathological criteria for diagnosing necrotizing soft tissue infections in patients undergoing triple diagnostics

Major histopathological criteria
Bullae
Severe fascia inflammation
Fascial necrosis

Minor histopathological criteria
Muscle edema
Severe muscle inflammation
Muscle necrosis
Capillary thrombosis
Vasculitis

**Table 4:** Key points for using frozen sections for diagnosing necrotizing soft tissue infections

Key points
Obtain full-thickness (epidermis to muscle) incisional biopsy; in contrast to the biopsy for microbiological evaluation, which must consist of only the fascia.
Report from which location, in relation to the infection, the incisional biopsy was taken
Only order frozen section assessment if results will have acute consequences for treatment strategy (e.g. not if evident macroscopic fascial necrosis is seen, patient will undergo amputation regardless of results, patient is deceased). Otherwise, order histopathologic assessment as standard formalin fixed paraffin embedded sample
Be aware of possible loss of subcutaneous fat during processing of frozen section
Use frozen sections as part of the triple diagnostic principle, not as independent test. Frozen sections make the diagnosis more or less likely, but do not diagnose the necrotizing soft tissue infection

The histopathological criteria regarding the fascia found in this study are comparable with prior studies (Table 5) [10, 13]. Although microscopic fascial necrosis was found to be a predictive finding for NSTIs in this current study and the study by Solomon et al. [13], two patients in our study had no microscopic fascial necrosis. In both cases, macroscopic fascial necrosis was reported in the operative report, resulting in hypothesis that these biopsies might have been taken from the resection margin (not well documented), and therefore, necrotic tissue was absent in the biopsy. Thus, it is important for surgeons to report the location from which area, in relation to the infection, the biopsy was taken. Furthermore, this shows that only looking for (microscopic) fascial necrosis does not result in 100% sensitivity, and therefore, biopsies should be assessed for other, additional, histopathologic findings. Considering that histopathological findings in the fascia as well as in the epidermis and muscle were predictive, obtaining full-thickness biopsies is strongly recommended. Unfortunately, only 21% of the biopsies in this study were full-thickness biopsies, so awareness among surgeons to obtain full-thickness biopsies (epidermis to muscle) should increase [6, 10, 11]. This is in contrast to samples for Gram-staining and culture, which preferable only contains clean handled fascia [7]. This is the first study differentiating the histopathological findings by tissue layer for NSTIs [10, 13].

**Table 5:** Literature overview of histopathological findings for diagnosing necrotizing soft tissue infections

Authors	Most important histopathological finding
Stamenkovic et al. [11]	Intact superficial dermis and epidermis
	Within superficial fascia, deep dermis and surrounding adipose tissue:
	Necrosis
	Polymorphonuclear infiltration
	Microorganisms
	Vasculitis
	Thrombosis
Stegeman et al. [10]	Whole microscopic view filled with granulocytes in the subcutis and fascia
Solomon et al. [13]	More severe inflammation
	More extensive necrosis
	Presence of bacteria
	Presence of karyorrhexis
	Presence of fibrine

Even if multiple tissue layers are biopsied, there is still a risk of losing tissue layers during processing of frozen sections. In 32% of the biopsies, tissue layers were absent in the frozen section compared to the FFPE sample, which was most often the subcutaneous fat. This is commonly caused by sampling errors; fat needs lower temperature to freeze compared to most other tissues, but when fat is overfreezed, it shatters more easily. Furthermore, fatty tissues are known to be more difficult to cut which can cause artifacts [15, 16]. Due to the instability of subcutaneous fat in frozen sections, subcutaneous fat findings were not included in our criteria.

In 38% of the biopsies, there were histopathological discrepancies between the frozen section and the FFPE sample. The signs of NSTIs appeared to be less outspoken in the frozen section (e.g. less severe inflammation, absent thrombi). The discrepancies in this study were most commonly due to the assessment of more superficial slices from the incisional biopsy for the frozen section and not due to sampling errors or artifacts. Even though discrepancies between frozen sections and FFPE samples were common and could result in underestimation of certain histopathologic characteristics, the diagnosis made and corresponding treatment strategy based on the frozen sections did not change after assessment of the FFPE sample. This is in line with studies from other medical fields using frozen sections, which report low rates of discordance between the frozen section conclusion and final diagnosis [14, 17, 18].

Histopathologic assessment is beneficial for diagnosing NSTIs, since clinical symptoms are not seldom unreliable [4, 5, 19]. A prior study showed that macroscopic fascial necrosis during surgical exploration has a high positive predictive value, but a low negative predictive value and therefore cannot simply rule out NSTIs [7]. Especially patients without evident macroscopic fascial necrosis benefit from using frozen sections, since half of the patients with NSTIs in this study did not have any signs of macroscopic fascial necrosis, while microscopic necrosis was (already) present. Also, patients with a high clinical suspicion for NSTI but with eventually a non-NSTI benefited from the frozen section, since ruling out microscopic necrosis in case of per-operative fascial edema averted unnecessary debridement. However, there were two patients with non-NSTIs and microscopic fascial necrosis in this study. A NSTI was eventually very unlikely in both cases based on the macroscopic evaluation, a negative Gram stain and the whole histological image. In these patients, it can be argued if a frozen section should have been taken in the first place, since the fascia was vital and no dishwater fluid or edema (which was present in all other ambivalent cases) was found upon macroscopic assessment. This stresses using frozen sections as part of the triple diagnostics algorithm and not as independent test. Using frozen sections in combination with other tests increases the reliability of the diagnosis made, especially in ambivalent cases.

Imaging, such as plain radiographs or computed tomography (CT), has also been proposed for diagnosing (ambivalent) NSTIs; however, it is not part of the diagnostic work-up at our institute and remains controversial in the current literature [8]. The use of CT-scans might be beneficial for certain patients, such as patients with a suspected intra-abdominal source of the infection [20]. A few studies have evaluated the diagnostic value of CT scans, all reporting high specificity, but widely variable sensitivity results [21–23]. Martinez et al. [22] proposed diagnosing NSTIs by assessing CT-scans using four criteria, resulting in a sensitivity of 100% and specificity of 98%. On the other hand, McGillicuddy et al. reported a sensitivity of 43% for diagnosing NSTI based on fascial air (specificity 98%) and a sensitivity of 39% for diagnosing NSTIs based on fluid tracking on CT-scans (specificity 85%), both criteria (with low sensitivity in the literature) were used in the criteria by Martinez et al. [22, 23]. Furthermore, CT scans cannot be performed in hemodynamically unstable patients (which is upon presentation frequent the case in NSTI patients) and could cause a significant delay in the surgical treatment of NSTIs [24, 25].

This study is limited by its small sample size, which constrained us to a descriptive analysis. One of the biggest arguments made against using frozen sections for diagnosing NSTIs is that it requires a pathologists with frozen section expertise [8]. Fortunately, we had the benefit of an experience pathologist who specializes in skin and soft tissues to re-assess all samples. With our protocolled assessment, we aimed to simplify the assessment, maintain generalizability and applicability of our results, and provide some sort of quantitative assessment of the samples and thus recommendations. During the retrospective scoring and assessment of the samples by the pathologist, the conclusion made did not differ from the conclusion reported in the first pathology report. The retrospective assessment only resulted in more details. We acknowledge that the implementation of frozen sections for diagnosing NSTI is affected by a hospital resources and regional collaborations, which requires the appropriate logistics for assessing frozen sections in emergency setting.

## Conclusion

The presence of bullae, severe fascial or muscle inflammation, fascial or muscle necrosis, muscle edema, thrombosis or vasculitis upon histopathological evaluation all indicate a high probability of a NSTI. In more than half of all surgical explorations for NSTIs, frozen sections aided in making the diagnosis NSTI more or less likely. Even though discrepancies between frozen and FFPE samples were common, none of the diagnoses made based on the frozen section had to be revised after examination of the FFPE samples. Therefore, we recommend frozen section for diagnosing NSTIs in ambivalent cases as part of the triple diagnostics algorithm.

## Figures and Tables

**Table 6:**
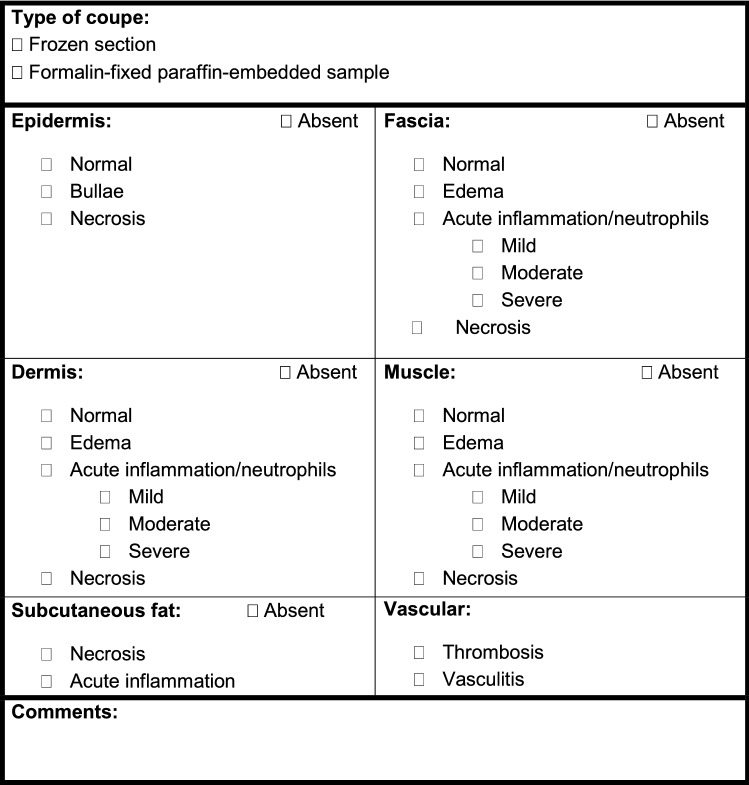
Assessment form for assessing frozen sections and formalin-fixed paraffin-embedded samples for signs of a necrotizing soft tissue infection.

## Appendix 1

See Table 6.

## Appendix 2

See Table 7.

**Table 7:** Histopathological characteristics of incisional biopsies from patient with eventually no necrotizing soft tissue infection

Tissue layer	Cellulitis (*n* = 5)	Erysipelas (*n* = 5)	Bacteremia (*n* = 1)	ADR with cutaneous eosinophilia (*n* = 1)
Epidermis (*n* = 6)				
No abnormalities	2/2	2/2	1/1	0/1
Bullae	0/2	0/2	0/1	0/1
Necrosis	0/2	0/2	0/1	1/1
Dermis (*n* = 6)				
No abnormalities	0/2	0/2	1/1	0/1
Edema	2/2	2/2	0/1	1/1
Acute inflammation				
Mild	1/2	2/2	0/1	0/1
Moderate	1/2	0/2	0/1	0/1
Severe	0/0	0/2	0/1	0/1
Necrosis	0/2	0/2	0/1	0/1
Subcutaneous fat (*n* = 11)				
Inflammation (regardless of severity)	3/4	3/5	1/1	0/1
Necrosis	2/4	2/5	1/1	0/1
Fascia (*n* = 12)				
No abnormalities	0/5	2/5	1/1	0/1
Edema	4/5	3/5	0/1	1/1
Acute inflammation				
Mild	3/5	2/5	0/1	0/1
Moderate	1/5	0/5	0/1	1/1
Severe	0/5	0/5	0/1	0/1
Necrosis	1/5	0/5	0/1	1/1
Muscle (*n* = 7)				
No abnormalities	0/2	2/4	1/1	NA
Edema	0/2	0/4	0/1	NA
Acute inflammation				
Mild	2/2	1/4	0/1	NA
Moderate	0/2	0/4	0/1	NA
Severe	0/2	0/4	0/1	NA
Necrosis	0/2	1/4	0/1	NA
Vessels (*n *= 12)				
Thrombosis	0/5	0/5	0/1	1/1
Vasculitis	0/5	0/5	0/1	0/1

ADR = adverse drug reaction; NA = not available

## Abbreviations

AUC: Area under the curve
ADR: Adverse drug reaction
FFPE: Formalin-fixed paraffin-embedded
NSTI: Necrotizing soft tissue infection
ROC: Receiver operative characteristics
